# Knowledge and Practice of Foot Care among Patients with Diabetes Attending Diabetes Center, Saudi Arabia

**DOI:** 10.3390/healthcare12131244

**Published:** 2024-06-22

**Authors:** Mona Eihab Aljaouni, Adel Mouad Alharbi, Omar M. Al-Nozha

**Affiliations:** 1 Ministry of Health, Madinah 42351, Saudi Arabia; maljaouni@moh.gov.sa (M.E.A.); dr.adelalhrbi@gmail.com (A.M.A.); 2 Medicine Department, College of Medicine, Taibah University, Madinah 42353, Saudi Arabia

**Keywords:** diabetes mellitus, diabetic foot, knowledge, foot care practice, Saudi Arabia

## Abstract

**Background:** Diabetic foot is a serious and often debilitating diabetes complication that poses a significant risk of morbidity and even mortality. However, ensuring good knowledge and good practice of appropriate foot care for patients with diabetes has been frequently neglected in diabetes management. **Objectives:** This study aimed to assess foot care knowledge and practice in patients with diabetes. **Methods:** We conducted a cross-sectional study on 400 patients with diabetes at Madinah Diabetes Center, Madinah City, Saudi Arabia, in 2023. Sociodemographic, knowledge score, practice of foot care, and diabetes-related data were collected using a valid interview structured questionnaire. The prevalence of good knowledge and practice level was calculated and compared using the studied patients’ characteristics using appropriate statistical tests. **Results:** The prevalence of good knowledge of foot care and its practice was 35% and 27%, respectively. The knowledge level showed statistically significant differences among patients based on their age and diabetes type and duration. Patients who were >50 years (70.1%), had type 2 diabetes (89.5%), and with diabetes duration >10 years (65%) showed significantly better knowledge. Female patients (65.7%) had a higher good practice level compared with male patients (34.3%) (*p* < 0.001). **Conclusions:** This study highlights the insufficient knowledge and inadequate foot care practice among patients with diabetes in the studied population. Educational interventions and targeted strategies are necessary to improve knowledge about the importance of foot care and promote better foot care practices among patients with diabetes.

## 1. Introduction

Diabetes mellitus (DM) is a global public health problem with a high burden worldwide [1]. Approximately 537 million individuals between the age of 20 and 79 years live with diabetes (1 in 10). This is expected to rise even further to reach 643 million by the year 2030, and by 2045, it is predicted that around 783 million people will be living with the disease if no actions are taken to reduce the prevalence [2]. In the Middle East, approximately 81 million individuals have T2DM, with Kuwait, Saudi Arabia, and UAE having the highest number of T2DM (>21%) [3]. The World Health Organization (WHO) reported that the prevalence of T2DM in Saudi Arabia is the second highest in the Middle East and the seventh highest globally [4]. The estimated number of individuals in Saudi Arabia living with diabetes exceeds 7 million, with an additional 3 million living with pre-diabetes. This increased prevalence led to a significant increase in healthcare expenses in the past two decades [5].

T2DM is associated with both short- and long-term complications that are of great concern. Long-term complications can be categorized mainly as either microvascular or macrovascular in nature, and many of these complications could be life-threatening. These include retinopathy, neuropathy, and nephropathy (microvascular), in addition to peripheral artery disease, cardiovascular disease, and cerebrovascular diseases (macrovascular). These complications pose significant risks to the health and well-being of individuals with T2DM [6,7]. Patients with uncontrolled T2DM will have an increased incidence of these complications, resulting in more hospitalizations, more deaths, and poorer quality of life. Thus, glycemic control and achieving near-normal glycemia as early as possible after type 2 diabetes is diagnosed is essential in minimizing the lifetime risk of diabetes-related complications [8]. 

Based on the recent literature, the prevalence of diabetic peripheral neuropathy (DPN) among diabetes patients in Saudi Arabia is 39% [9]. This prevalence is similar to the rates reported in the UK (32%) [10] and Italy (31%) [11], but relatively lower than Turkey (60%) [12], Iran (49%) [13], the USA (45%) [14], and China (62%) [15]. DPN can lead to painful peripheral neuropathy and diabetic foot ulceration [16,17,18]. Amputation can occur in one in four patients with diabetes with foot ulcerations, and their risk of mortality is 2.5 times higher than those without foot ulcers [16].

The majority of foot issues can be avoided with careful foot maintenance and self-care. Good foot care habits may take some time and effort to develop, but self-care is crucial [19]. Several prior studies have shown that diabetes patients’ knowledge of and adherence to proper foot care practices reduce their risk of foot ulcerations and amputation. Chellan et al. [19] and Saurabh et al. [20] have reported an inverse relationship between the knowledge about proper foot care, its practice, and foot ulcerations. Moreover, the importance of diabetes patients’ knowledge of and adherence to proper foot care practices has also been highlighted [21,22]. Overall, diabetic foot complications pose a significant burden on diabetes patients, leading to morbidity, mortality, and healthcare costs. Proper foot care practices indeed play a crucial role in preventing and managing these complications [19,20,21,22]. However, previous studies have shown that patients with diabetes often have inadequate knowledge and adherence to foot care practices. Moreover, ensuring good knowledge and good practice of appropriate foot care for patients with diabetes have been frequently neglected in diabetes management. Accordingly, we aimed to assess the knowledge and practice of foot care among diabetic patients attending the outpatient clinics at Madinah Diabetes Center (MDC). 

## 2. Methods

We conducted a descriptive cross-sectional study, which included patients with diabetes at MDC, to evaluate their knowledge and practice toward foot care. MDC is a part of King Fahd Hospital (KFH) in Madinah City, which is one of the largest governmental specialized centers providing healthcare services to the local population and visitors. MDC serves diabetes patients and offers comprehensive services, including ophthalmology, foot care, education, and nutrition clinics. The center has served 16,678 diabetic patients. We enrolled all diabetic patients, both Saudi and non-Saudi, who visited MDC from July 2023 to January 2024. Eligible participants included adult diabetic patients (types 1 and 2) aged ≥15 years. We excluded those aged less than 15 years, patients with gestational diabetes, and those who declined to participate. 

The sample size was determined using the Epi-Info program, considering previous Saudi studies reporting knowledge of foot care of 55.1% [23,24], an alpha error of 0.05, and a confidence limit of 95%. The estimated sample size was 372, and ultimately, 400 patients participated and were analyzed.

We used simple convenience sampling in selecting our study patients. Diabetes patients visiting diabetes clinics who consented to participate and were not excluded according to the exclusion criteria were interviewed by a family medicine physician and a trained medical student using a predesigned structured Arabic questionnaire, which was translated from a previously used tool as part of the Diabetes Care Program of Nova Scotia [25]. It was translated into Arabic and then backtranslated into English to ensure accuracy. The Arabic version of the questionnaire we utilized underwent cross-cultural adaptation to align with local traditions and habits. A pilot study was performed on 10 participants. No changes were made to the tool after that, and the results were not included in the current study.

The study questionnaire collected sociodemographic and identification data, including age (≤50 and >50 years), sex (male and female), nationality (Saudi and non-Saudi), educational level (illiterate, less than secondary, secondary, university, and higher), occupation (student, employee, retired, and unemployment, including housewife), and smoking status (yes and no), as well as data related to diabetes mellitus (type and diabetes duration) and information on knowledge and practice about foot care, including history and current leg and foot problems, foot care, foot wear, safety and prevention, and foot care education (see Supplementary Materials).

After obtaining approval from the Ethical and Research Committee, the data were collected through face-to-face interview using the study questionnaire. Through the questionnaire, knowledge and practice were assessed.

### 2.1. Assessment of Knowledge

Foot care knowledge was evaluated using a set of eight questions with responses categorized as either “yes” or “no”. For each question, correct and incorrect answers were assigned a score of 1 and 0, respectively. The total knowledge score was calculated, and patients with scores of 0–5 were considered as having poor knowledge, and those who scored 6–8 were considered as having good knowledge.

### 2.2. Assessment of Practice

To evaluate practice, the patients were asked 15 questions to determine if they engaged in foot care practices. A score of 1 was assigned for each “yes” answer, and patients with scores of less than ten were considered as having poor practices and those with scores more than ten as having good practices. However, the last question regarding shoe type was not factored into this categorization.

### 2.3. Statistical Analysis

We entered and analyzed the collected data using the SPSS program, version 22. The data were presented using frequencies, means, and standard deviation for categorical and continuous variables, respectively. The studied patients’ knowledge and practice were assessed, analyzed, and compared using their identification data using chi-square and Fischer exact tests appropriately. Statistical significance was determined at *p* < 0.05. 

## 3. Results

The characteristics of the 400 diabetes patients are shown in Table 1. Among the participants, 60.8% were >50 years old, with an equal distribution of men and women. Most participants (92.5%) were of Saudi nationality. Approximately 22.3% of the patients were illiterate. In terms of educational background, 25.3% of the studied patients had less than secondary education, whereas 26% and 26.5% had completed secondary education and a university degree, respectively. Among the participants, 44.5% were unemployed, including 121 who were housewives. Most of the participants (81.7%) had T2DM, and >50% of the patients had been diagnosed with diabetes for >10 years. Additionally, a large proportion of the participants (85.8%) reported never having smoked. Among the studied patients, 58 (14.5%) previously experienced foot ulcers, whereas 14 (3.5%) underwent amputations involving their toes, foot, and/or leg. Moreover, 110 (27.5%) and 8 patients (2%) had foot calluses and foot ulcers accompanied by blisters, respectively.

Table 2 presents the complications related to diabetic foot among the studied patients, showing 58 (14.5%) and 14 patients (3.5%) had “previously experienced DFUs” and “undergone amputations involving their toes, foot, and/or leg”, respectively. Moreover, 31 (7.8%), 109 (27.3%), and 31 patients (7.8%) reported having “foot ulcers accompanied by blisters”, “foot calluses”, and “the presence of blood or discharge in their socks”, respectively. Additionally, 61.3% reported “numbness, tingling, pins and needles, or itching sensation in their feet”, and 44.5% reported “tightness, heaviness, pain, or cramps in their feet or legs”.

Table 3 presents the responses of all the patients regarding their knowledge of foot care. Among the participants, 170 (42.5%) were aware that individuals with diabetes can develop foot ulcers. Additionally, 100 patients (25%) knew that they may experience decreased sensation in their feet. Moreover, 160 respondents (40%) acknowledged the risk of developing foot ulcers. Furthermore, 44.5% (178 patients) were aware that they could develop foot gangrene, and 28.5% reported attending foot care classes. Furthermore, 20.2% stated that they obtained information from reading handouts on foot care, whereas 25.5% reported reading handouts specifically related to proper footwear. 

Table 4 presents the findings related to foot care practices of the included diabetic patients. Among the participants, 98% reported washing their feet daily, and 80.5% stated that they trim their toenails themselves. Furthermore, 70.3% of patients reported “testing the water temperature before putting their foot in”, and 79.3% reported “inspecting their shoes for foreign objects or torn linings” before wearing them. Regarding other foot care practices, 53%, 37.8%, and 58% of the patients reported walking barefoot, applying lotion or moisturizer between the toes, and sitting with crossed legs, respectively. However, 47% mentioned drying well between toes, 27% reported not wearing shoes without wearing any socks, and when they wore socks, they were made of cotton fabric (89.5%) and synthetics (10.3%), and only 1.8% wore diabetic socks. The shoe types varied and included sandals (49.3%), athletic shoes and sneakers (50.5%), pointed toes (2.5%), broad round shoes (3.8%), high knee (2.3%), and special/custom shoes (15.3%). Additionally, 3.2% of patients reported using medicated products to treat warts, corns, or calluses, whereas only 10.5% indicated “using a hot water bottle or heating pad on their feet”.

Table 5 presents the comparison of knowledge among the studied patients based on their characteristics. A good knowledge level showed statistically significant differences among the patients based on their age, occupation, and diabetes type and duration. A good knowledge level was significantly higher among patients aged >50 years (70.1%). Similarly, those who were not employed, including housewives, showed a higher proportion (45.7%) of good knowledge. Patients with T2DM exhibited a significantly higher percentage (89.5%) of good knowledge. Furthermore, patients with diabetes for >10 years (65%) had a good knowledge level. Although not statistically significant, some trends suggested higher proportions of good knowledge among men, Saudi nationals, individuals with secondary and university education, and non-smokers.

Table 6 shows the comparison of practice among patients based on their characteristics. The analysis revealed statistically significant differences in the level of good practice when considering the patient’s sex. Female patients exhibited a higher level of good practice compared with male patients, with proportions of 65.7% and 34.3%, respectively (*p* < 0.001). Although not reaching statistical significance, some trends indicate higher proportions of good practice among specific subgroups, including those who were unemployed (49.1%), had secondary and university education (52.8%), had T2DM (80.6%), had diabetes for >10 years (55.6%), and who never smoked (88.9%).

Table 7 shows the prevalence of good knowledge and foot practice among the studied patients regarding the presence of complications related to diabetic foot. Statistically, a significantly high proportion of good knowledge was found among patients who reported “numbness, tingling, pins and needles, or itching sensation in their feet” (82.9%), those who reported “tightness, heaviness, pain, or cramps in their feet or legs” (68.6%), and those with a “sore or cut on their foot or leg that healed after >2 weeks” (41.4%). The proportion of good knowledge was also high among patients who “did not experience foot ulcer” (85%), “amputation of a toe, foot, or leg” (95.7%), and “blood or discharge in their socks” (97.9%), although not significant. 

Table 7 displays the prevalence of good foot care practice among the studied patients by the presence of complications related to diabetic foot. Although not significant, a higher proportion of foot care practice was found among patients who “did not experience foot ulcer” (88.9%), “amputation” (97.2%), and “did not have an ulcer, sore, or blister on their feet” (91.7%), “did not have blood or discharge in their socks” (99.1%), and “had no callus on their feet” (74.1%).

## 4. Discussion

Our findings show a notable prevalence of inadequate knowledge and poor foot care practices among the participants. The individual knowledge about foot care did not exceed 50%. These results align with previous international studies reporting deficiencies in knowledge and practice regarding foot care among patients with diabetes [26,27,28]. A recent similar study conducted in Saudi Arabia on more than 400 adult patients with diabetes in Jazan showed that 56% of the patients had wrong answers to foot care knowledge questions [29].

The level of good knowledge in the present study was found in only 140 patients (35%), which was significantly different based on the studied patients’ characteristics.

A much lower level of good knowledge was reported in a recent study conducted in Makkah, Saudi Arabia, on just over 400 patients with diabetes attending Heraa Diabetes Center between June and July 2020. Most of the participants (72.4%) had a poor knowledge score, and only 4.2% had a good knowledge score [30]. Similarly, a recent cross-sectional study on 1080 patients with diabetes in North China reported that 25.1% had a low knowledge score of diabetes foot care and foot care practice [31]. By contrast, another Saudi study reported a good knowledge level of 55.1% [32]. 

Our study reports a significantly high knowledge level among patients aged ≥50 years, with 70.1% exhibiting good knowledge. Similarly, unemployed individuals, including housewives, had a higher proportion (45.7%) of good knowledge. Moreover, 89.5% of patients with T2DM had good knowledge. In contrast, another Saudi cross-sectional study conducted in Jeddah City, involving 747 patients with diabetes from seven primary health care centers, did not find a significant association between knowledge and sociodemographic variables, except for unemployed participants, where a significant association was observed [33]. In a study in Makkah, however, being employed, having T2DM, and relying on health staff as the primary source of information were factors significantly associated with a higher knowledge level compared with their counterparts [30]. The inconsistency of these results might be attributed to variations in the study methodology, the questionnaire used, and patient characteristics.

Our findings show that most patients had negative responses for foot care practices, except for washing feet daily (97.5%), trimming toenails using file edges (72.5%), and inspecting shoes for torn linings before wearing them (79.2%). Except female sex (65.7%), the practice of foot care did not show significant differences based on patient characteristics. The foot care practice in a Chinese study was high, wherein many of the studied patients reported treating corns, calluses, and wounds on their own [30]. The practice of foot care was reported as good in >80% of the participants, but some areas had poor practices [30]. A cross-sectional study conducted throughout Saudi Arabia on 519 patients with diabetes reported that 56.5% of the participants scored between 6 and 10 out of 15 in the evaluation of practice of foot care [34].

In the present study, nonsmoker patients had a higher knowledge level and better practice regarding foot care. This finding was consistent with a study conducted in China [30], which reported that smokers had lower knowledge levels and poorer attitudes toward foot care compared with nonsmokers. Several possible explanations can account for this result. One contributing factor may be lower health literacy among smokers and being reluctant to accept new information related to their diseases, and such factors could make the understanding of mechanisms of their disease and the methods for preventing associated complications very challenging [35]. Additionally, patient sex was an important barrier. Women tend to be more attentive to their diseases signs and symptoms and are more concerned about their body image. In contrast, men often hesitate to acknowledge having a health problem and, furthermore, to seek professional care [36]. Moreover, many men do not seek proper medical attention even after acknowledging that they have been diagnosed with a chronic disease, probably due to their time constraints and the mismatch between their working hours and health clinic hours [37]. A meta-analysis examining amputation risk factors in patients with diabetic foot found that the pooled risk for amputations was 1.5 times higher for men [38].

For other patients’ characteristics, several studies have examined the association between education level, knowledge scores, and practice of foot care among patients with diabetes. These studies consistently found that patients with lower education levels had less knowledge about foot care compared with their counterparts [29,32,33]. Patient education plays a crucial role in enhancing knowledge of appropriate foot care, which reduces the risk of foot ulceration and amputation in high-risk diabetic individuals [39]. The link between education and knowledge of foot care may be attributed to educated patients being more health-conscious, having easier access to educational resources, and using technology to gather information about their condition [40]. Patients with a longer diabetes duration had significantly greater knowledge. This may be attributed to patients accumulating more information and experience over time. However, these findings contradict those of other studies, which reported that diabetes duration does not impact a patient’s knowledge of the disease [41].

This study’s findings reveal that 58 (14.5%) and 14 patients (3.5%) had DFU and underwent “amputations involving their toes, feet, and/or legs”, respectively. Moreover, 31 patients (7.8%) reported “foot ulcers accompanied by blisters”, whereas 109 patients (27.3%) reported “foot calluses”, and 31 patients (7.8%) reported “having blood or discharge in their socks”. Various studies conducted in Saudi Arabia have explored the prevalence of diabetic foot ulcers and show varying results ranging from 26.0% to 61.8% [31,32]. The prevalence in the region of Qassim, Saudi Arabia, of such ulcers was 10.8%, and the prevalence of amputation of a toe, foot, or an entire leg was 2.5% [42]. In terms of foot care practices, Goweda et al. [43] reported that 77.1% of patients examined their feet. With regard to foot care knowledge, 49.1% of the participants received a handout on foot care [43]. A previous report by the Ministry of Health (MOH) in Saudi Arabia showed that diabetic foot was a major cause of lower limb amputation in Saudi Arabia [44]. 

The study findings have also shown that 61.3% of patients had “numbness, tingling, pins and needles, or itching sensations in their feet”, and (44.5%) had “tightness, heaviness, pain, or cramps in their feet or legs”. All these manifestations are the presenting symptoms of diabetic peripheral neuropathy (DPN). The prevalence of DPN is the most prevalent diabetic complication, which can lead to significant morbidities, including painful DPN and DFU [16,17,18]. The proportion of good knowledge was high among patients who did experience a foot ulcer (85%), “amputation of a toe, foot, or leg” (95.7%), and did not report “blood or discharge in their socks” (97.9%), although not significant. Additionally, a higher proportion of foot care practice was found among patients who did not experience a foot ulcer (88.9%), amputation (97.2%), and did not have ulcers, sores, or blisters on their feet (91.7%), did not have blood or discharge in their socks (99.1%), and had no callus (74.1%). Consistent with these findings, the results of previous similar studies reported that diabetic patients without diabetes complications had significantly higher knowledge and better foot care practice than those with complications [29,44,45]. These findings were consistent with the results by Guell et al. [46], who reported that lower knowledge and practice scores, as expected, might make patients more vulnerable to complications.

### Study Strengths and Limitations 

In our study, we have analyzed the problem in relation to different sociodemographic and diabetes-related factors. Moreover, a face-to-face interview questionnaire during the data collection validated the information gathered for the analysis. In addition, the use of structured and validated questionnaires has facilitated confidence in obtaining sound information in a standardized manner. However, our study has its own limitations. Causality of association cannot be established in such a cross-sectional design. In addition, although the study was conducted in the largest diabetes center in Madinah, serving both Madinah and nearby regions, our study findings cannot be generalized to all diabetes patients in Saudi Arabia. However, the main scope of this study was to shed light on this important public health issue and to trigger more future in-depth research, hopefully on a national level.

## 5. Conclusions

The present study highlights the insufficient diabetes patients’ level of knowledge and poor practice of proper foot self-care in the studied population. These results should serve as a crucial alert for clinicians, nurses, and policy makers in public health authorities to emphasize the importance of implementing patient-centered and physician-friendly educational programs to enhance and sustain good and proper foot self-care knowledge and practice among patients with diabetes. We believe that future educational programs should focus more on improving the attitude of patients toward their personal foot care and on practicing personal foot care in an educational activity (practical sessions), as this could have more impact on the sustainability of learned information about personal foot care than traditional educational material.

## Figures and Tables

**Table 1:** Characteristics of studied patients.

Characteristics	N = 400
Age in years	
≤50	157 (39.2)
>50	243 (60.8)
Sex	
Male	200 (50.0)
Female	200 (50.0)
Nationality	
Saudi	371 (92.5)
Non-Saudi	29 (7.5)
Educational level	
Illiterate	89 (22.3)
Less than secondary	101 (25.3)
Secondary	104 (26.0)
University and higher	106 (26.5)
Occupation	
Student	38 (9.5)
Employee	78 (19.5)
Retired	105 (26.3)
Unemployment *	179 (44.8)
Type of DM	
Type 1	73 (18.3)
Type 2	327 (81.7)
Duration of diabetes	
<5 years	67 (16.8)
5–10 years	123 (30.8)
>10 years	210 (52.5)
Smoking	
No	344 (85.8)
Yes	57 (14.2)

* Including 121 housewives.

**Table 2:** Prevalence of complications related to diabetic foot among the studied patients.

Complications Related to Diabetic Foot	Yes n (%)	No n (%)
Have you ever had a sore or cut on your foot or leg that took more than 2 weeks to heal?	70 (17.5)	330 (82.5)
Have you ever had a foot ulcer?	58 (14.5)	342 (85.5)
Have you ever had an amputation of a toe, foot, or leg?	14 (3.5)	386 (96.5)
Do you have an ulcer, sore, or blister on your feet at this time?	31 (7.8)	369 (92.2)
Do you have blood or discharge in your socks?	10 (2.5)	390 (97.5)
Do you have calluses on your feet?	109 (27.3)	291 (72.7)
Do you have any numbness, tingling, pins and needles, or itching sensation in your feet?	245 (61.3)	155 (38.7)
Do you have any tightness, heaviness, pain, or cramps in your feet or legs?	178 (44.5)	222 (55.5)

**Table 3:** Patients responses regarding their knowledge of foot care.

Knowledge Items	Yes n (%)	No n (%)
Do you know that diabetics can develop foot ulcers?	170 (42.5)	230 (57.5)
Do you know that diabetics may develop lack of sensation in their feet?	100 (25.0)	300 (75.0)
Do you know that loss of sensation in the feet makes them prone to foot ulcers?	160 (40.0)	240 (60.0)
Do you know that reduced blood flow to the feet makes them prone to foot gangrene?	178 (44.5)	222 (55.5)
Have you ever attended a class on how to care for your feet?	114 (28.5)	286 (71.5)
Do you ever read any handouts on foot care?	81 (20.3)	319 (79.7)
Have you read handouts on proper footwear?	102 (25.5)	298 (74.5)
Do you have a handout on how to care for your feet?	273 (68.3)	127 (31.7)

**Table 4:** Practice of foot care among the studied patients.

Practice Items	Yes n (%)
Do you wash your feet every day?	392 (98.0)
Do you dry well between your toes?	188 (47.0)
Do you cut your toenails by yourself?	322 (80.5)
Do you always test the water temperature before putting your foot in?	281 (70.3)
Do you use medicated products for warts, corns, or calluses?	13 (3.2)
Do you use moisturizing cream on your feet?	229 (57.3)
Do you put moisturizing creams or lotions between your toes?	151 (37.8)
Do you ever walk around in your bare feet?	212 (53.0)
Do you wear shoes without wearing any socks?	292 (73.0)
Do you always inspect your shoes for foreign objects or torn linings?	317 (79.3)
Do you use a hot water bottle or heating pad on your feet?	42 (10.5)
Do you ever soak your feet?	109 (27.3)
Do you examine your feet every day?	105 (26.3)
Do you sit with your legs crossed?	232 (58.0)
What type of shoes do you wear?	
Sandals	197 (49.3)
Athletic shoes and sneakers	202 (50.5)
Pointed toe	10 (2.5)
Broad and round	15 (3.8)
High knee	9 (2.3)
Special/custom shoes	61 (15.3)

**Table 5:** Comparison of knowledge among the studied patients based on their characteristics.

Characteristics		Good Knowledge (n = 140)	Poor Knowledge (n = 260)	*p* Value
Age in years	≤50	39 (27.9)	118 (45.4)	0.001 *
	>50	101 (70.1)	142 (54.6)	
Sex	Male	73 (52.1%)	127 (48.8)	0.52
	Female	67 (47.9)	133 (51.2)	
Nationality	Saudi	130 (92.9)	241 (92.7)	0.95
	Non-Saudi	10 (7.1)	14 (7.3)	
Education level	Illiterate	29 (20.7)	60 (23.0)	0.10
	Less than secondary	31 (22.1)	70 (26.9)	
	Secondary	34 (24.4)	70 (26.9)	
	University and higher	39 (27.8)	67 (25.2)	
Occupation	Student	6 (4.3)	32 (12.3)	0.03 *
	Employee	25 (17.9)	53 (20.4)	
	Retired	45 (32.1)	60 (23.1)	
	Unemployed *	64 (45.7)	115 (44.2)	
DM Type	Type 1	15 (10.7)	58 (223)	0.004 *
	Type 2	125 (89.5)	202 (77.7)	
Diabetes duration	<5 years	15 (10.7)	52 (20.0)	0.001 *
	5–10 years	34 (24.3)	89 (34.2)	
	>10 years	91 (65.0)	119 (45.8)	
Smoking	No	124 (88.6)	220 (84.6)	0.27
	Yes	16 (11.4)	40 (15.4)	

* Significant.

**Table 6:** Comparison of practice among the studied patients based on their characteristics.

Characteristics		Good Practice (n = 108)	Poor Practice (n = 292)	*p* Value
Age in years	≤50	45 (41.7)	112 (38.4)	0.54
	>50	63 (58.3)	180 (61.6)	
Sex	Male	37 (34.3)	163 (55.8)	<0.001 *
	Female	71 (65.7)	129 (44.2)	
Nationality	Saudi	103 (95.4)	268 (91.8)	0.21
	Non-Saudi	5 (4.6)	24 (8.2)	
Education level	Illiterate	19 (18.0)	70 (23.6)	0.42
	Less than secondary	21 (19.2)	80 (27.3)	
	Secondary	26 (24.1)	78 (26.7)	
	University and higher	30(28.7)	76(26.4)	
Occupation	Student	9 (8.3)	29 (9.9)	0.71
	Employee	21 (19.4)	57 (19.5)	
	Retired	25 (23.1)	80 (27.4)	
	Unemployed *	53 (49.1)	126 (43.2)	
DM Type	Type 1	21 (19.4)	52 (17.8)	0.70
	Type 2	87 (80.6)	240 (82.2)	
Diabetes duration	<5 years	16 (14.8)	51 (17.5)	0.73
	5–10 years	32 (29.6)	91 (31.2)	
	>10 years	60 (55.6)	150 (51.3)	
Smoking	No	96 (88.9)	248 (84.9)	0.30
	Yes	12 (11.1)	84 (14.1)	

* Significant.

**Table 7:** Prevalence of good knowledge and good foot care practice among the studied patients based on the presence of complications related to diabetic foot.

Complications Related to Diabetic Foot	Good Knowledge (n = 140) n (%)	*p* Value	Good Practice (n = 108) n (%)	*p* Value
Have you ever had a sore or cut on your foot or leg that took more than 2 weeks to heal?				
Yes	58 (41.4)	<0.001 *	21 (19.4)	0.55
No	82 (58.6)		87 (80.6)	
Have you ever had a foot ulcer?				
Yes	21 (15.0)	0.84	12 (11.1)	0.24
No	119 (85.0)		96 (88.9)	
Have you ever had an amputation of a toe, foot, or leg?				
Yes	6 (4.3)	0.53	3 (2.8)	0.76
No	134 (95.7)		105 (97.2)	
Do you have an ulcer, sore, or blister on your feet at this time?				
Yes	28 (20.0)	<0.001 *	9 (8.3)	0.79
No	112 (80.0)		99 (91.7)	
Do you have blood or discharge in your socks?				
Yes	3 (2.1)	1.00	1 (0.9)	
No	137 (97.4)		107 (99.1)	0.30
Do you have calluses on your feet?				
Yes	31 (22.1)	0.10	28 (25.9)	0.71
No	109 (77.9)		80 (74.1)	
Do you have any numbness, tingling, pins and needles, or itching sensation in your feet?				
Yes	116 (82.9)	<0.001 *	69 (63.9)	0.51
No	24 (17.1)		39 (36.1)	
Do you have any tightness, heaviness, pain, or cramps in your feet or legs?				
Yes	96 (68.6)	<0.001 *	54 (50.0)	0.18
No	44 (31.4)		54 (50.0)	

* Significant.

## Data Availability

The original contributions presented in this study are included in the article; further inquiries can be directed to the corresponding authors.

## Supplementary Materials

The following supporting information can be downloaded at: https://www.mdpi.com/article/10.3390/healthcare12131244/s1, Diabetes Foot Care Questionnaire.
