# Supplementary material for: Knowledge and Practice of Foot Care among Patients with Diabetes Attending Diabetes Center, Saudi Arabia
Source: Healthcare (Basel). 2024 Jun 22;12(13):1244. doi: 10.3390/healthcare12131244 (PMC11240979; doi:10.3390/healthcare12131244)
Supplement: Supplementary file 1 [file healthcare-12-01244-s001.zip › healthcare-3034975-supplementary.pdf]

Diabetes Foot Care Questionnaire

This research is carried out by Dr. Mona Aljaouni & Dr.Adel Alharbi (diabetes fellow) to complete the requirement of obtaining the Saudi specialization in diabetes.

Which is entitled :

Knowledge and Practice Of Foot Care Among Diabetic Patients Attending Diabetic Center In King Fahad Hospital, Madinah , Saudi Arabia, 2023.

Please read the questions carefully before answering. Your answers will be kept confidential.

Filling this questionnaire considers a written consent . Thank you for your cooperation.

- Do you accept to fill in this questionnaire?
- Yes
- No

Personal data:

1-Name: (optional)   ……………………………

2-Gender: □ Female   □ Male

3-Age: ……….   year

4-Nationality : □ Saudi   □ non-Saudi

5-Profession:……………..

Taking care of your feet is an important part of diabetes care. Please answer the following questions about your feet and how you care for them. Please return the completed form to the Diabetes Centre.

History of Foot Problems

- Have you ever had a sore or cut on your foot or leg that took more than two weeks to heal? ❑ Yes ❑ No
- Have you ever had a foot ulcer? ❑ Yes ❑ No
- Have you ever had an amputation of a toe, foot, or leg? ❑ Yes ❑ No

(If yes, date: _______/______/______).

Current Foot or Leg Problems

- Do you have an ulcer, sore, or blister on your feet at this time? ❑ Yes ❑ No
- Do you have blood or discharge on your socks? ❑ Yes ❑ No
- Do you have any calluses on your feet? ❑ Yes ❑ No
- Do you have any numbness, tingling, pins and needles, or itching sensation in your feet? ❑ Yes ❑ No
- Do you have any tightness, heaviness, pain, or cramps in your feet or legs? ❑ Yes ❑ No

---

Foot Care

- Can you reach and ***see*** the bottoms of your feet? ❑ Yes ❑ No
- Do you examine your feet? (If yes, how often?) ❑ Yes ❑ No

❑ Every day □ 2-6 times a week

❑ Once a week or less □ When I have a problem

- Do you wash your feet everyday? ❑ Yes ❑ No
- Do you dry well between the toes? ❑ Yes ❑ No

- Do you use a moisturizing cream on your feet? ❑ Yes ❑ No
- Do you cut your own toenails? (If no, who does this for you?) ❑ Yes ❑ No

□Family member □ Caregiver □ Foot care nurse □ Podiatrist

(complete other side)

Foot Wear

What kind of shoes do you wear? (Check all that apply.)

❑ pointed toes ▯ broad, round toes ▯ high heels

❑ sandals ▯ flip flops/thongs ▯ athletic/sneakers/runners

❑ shoes made of leather or canvas ▯ special/custom shoes

❑ shoes with adjustable laces, buckles or velcro

❑ What kind of socks do you wear? (Check all that apply.)

❑ cotton ❑ wool ❑ acrylic/synthetic

❑ knee highs ❑ elastic-free tops ❑ seamless socks

❑ nylons/pantyhose ❑ "diabetes" socks ❑ prescription/compressio

---

Safety and Prevention

- Do you ever soak your feet? ❑ Yes ❑ No
- Do you always test water temperature before putting your foot in? ❑ Yes ❑ No
- Do you use medicated products for warts, corns or calluses? ❑ Yes ❑ No
- Do you put moisturizing creams or lotions between your toes? ❑ Yes ❑ No

---

- Do you ever walk around in your bare feet? ❑ Yes ❑ No
- Do you ever wear shoes without wearing any socks? ❑ Yes ❑ No
- Do you always inspect your shoes for foreign objects or torn linings? ❑ Yes ❑ No
- Do you use a hot water bottle or heating pad on your feet? ❑ Yes ❑ No
- Do you sit with your legs crossed? ❑ Yes ❑ No
- Do you smoke? ❑ Yes ❑ No

Foot Care Education

- Have you ever attended a class on how to care for your feet? ❑ Yes ❑ No
- Have you ever read any handouts on <u>foot care</u>? ❑ Yes ❑ No
- Have you ever read any handouts on proper <u>footwear</u>? ❑ Yes ❑ No
- Would you like a handout on how to care for your feet? ❑ Yes ❑ No

---

Thank you for completing this questionnaire!
